# Supplementary material for: Genome-scale analysis of interactions between genetic perturbations and natural variation
Source: Nat Commun. 2024 May 18;15:4234. doi: 10.1038/s41467-024-48626-1 (PMC11102447; doi:10.1038/s41467-024-48626-1)
Supplement: Supplementary file 1 — Supplementary Information [file 41467_2024_48626_MOESM1_ESM.pdf]

Supplementary information for

Genome-scale analysis of interactions between genetic perturbations and natural variation

Joseph J. Hale[1], Takeshi Matsui[2,#], Ilan Goldstein[1,#], Martin N. Mullis[1], Kevin Roy[2], Chris Ne Ville[1], Darach Miller[2], Charlie Wang[1], Trevor Reynolds[1], Lars M. Steinmetz[2], Sasha F. Levy[2,%], Ian M. Ehrenreich[1,%]

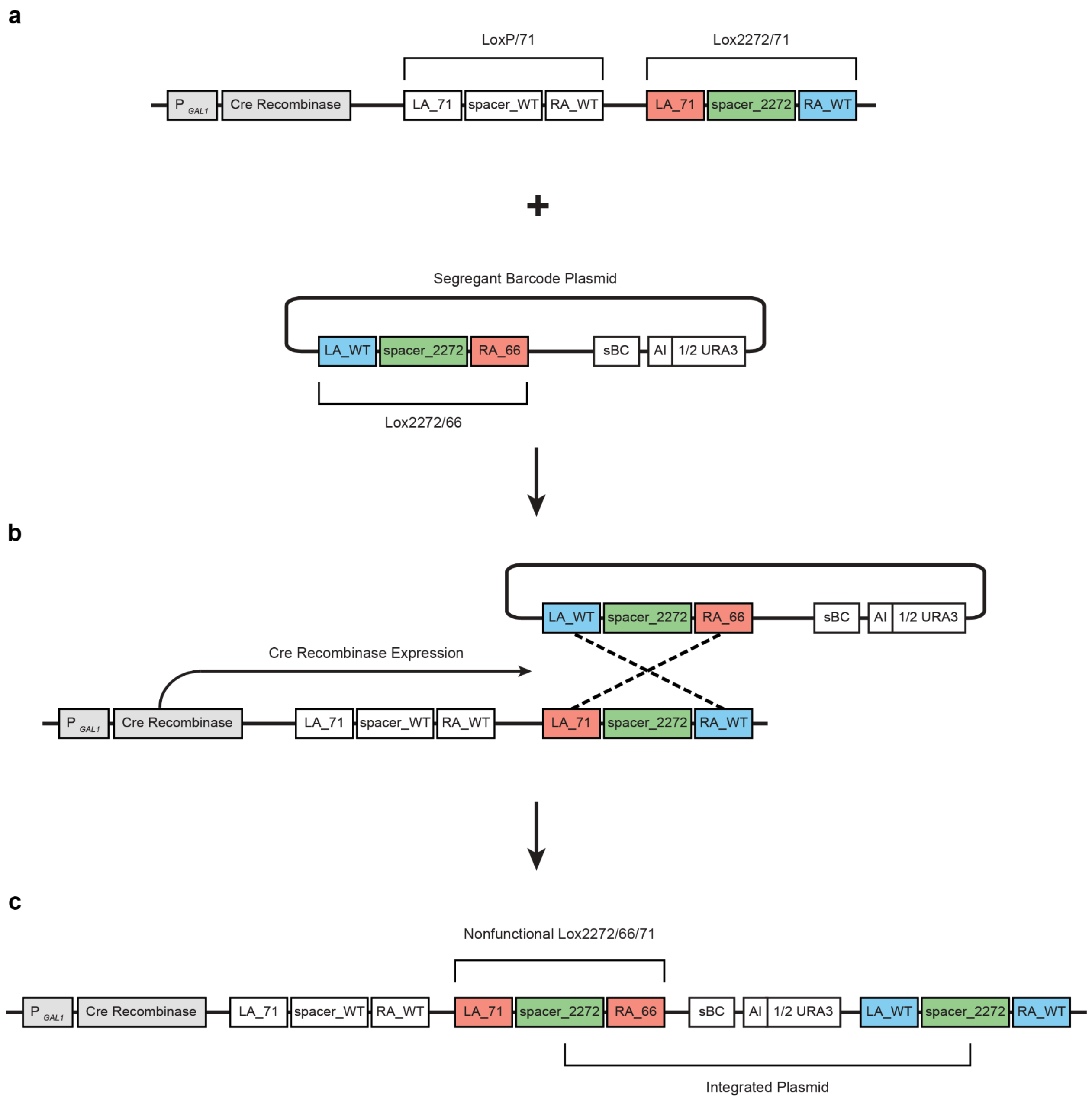

Supplemental Figure 1: Landing Pad design. a) Genomic landing pad before integration of any plasmids. LA_71 indicates a partial mutation on the left arm of the Lox site, which has no effect on its own. RA_66 indicates a similar partial mutation on the right arm. Lox2272/66 should only recombine with Lox2272/71. b) Expression of Cre recombinase causes the plasmid to be integrated into the genome in a predictable configuration. c) Recombination at the Lox2272 sites results in the plasmid contents

being integrated into the chromosome. This also places both mutant arms on the same Lox site, which renders it nonfunctional and limits spontaneous popout of the plasmid.

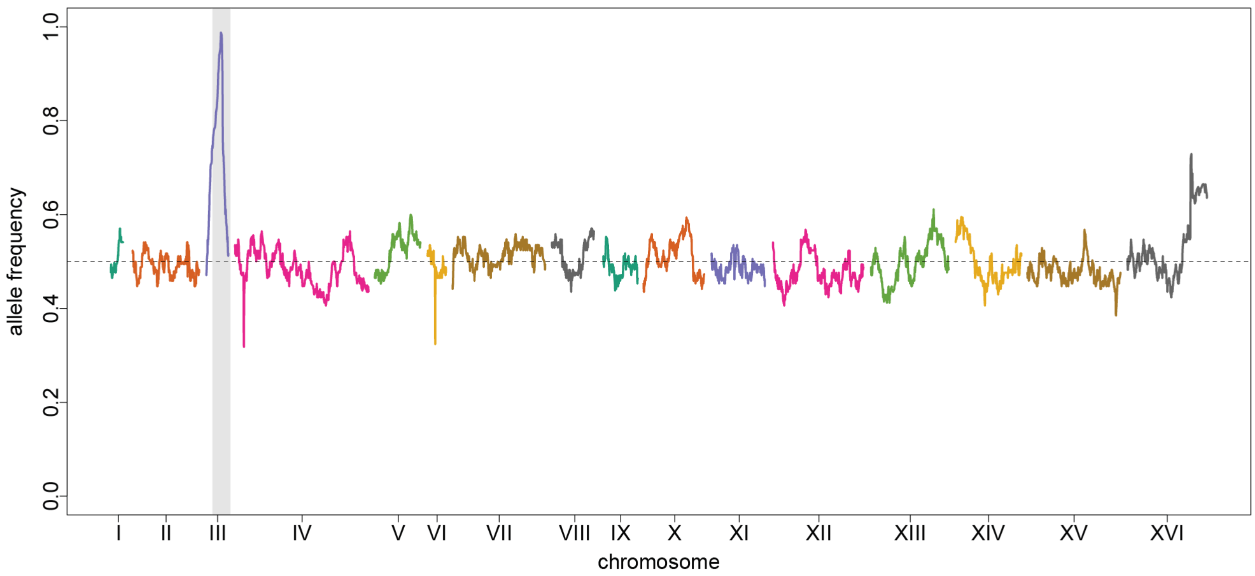

Supplemental Figure 2: Allele frequencies across all genotypes. The proportion of segregants carrying the 3S allele of a site is shown. The fixed region on Chromosome III is the mating locus, at which all segregants possess the 3S allele of MATalpha.

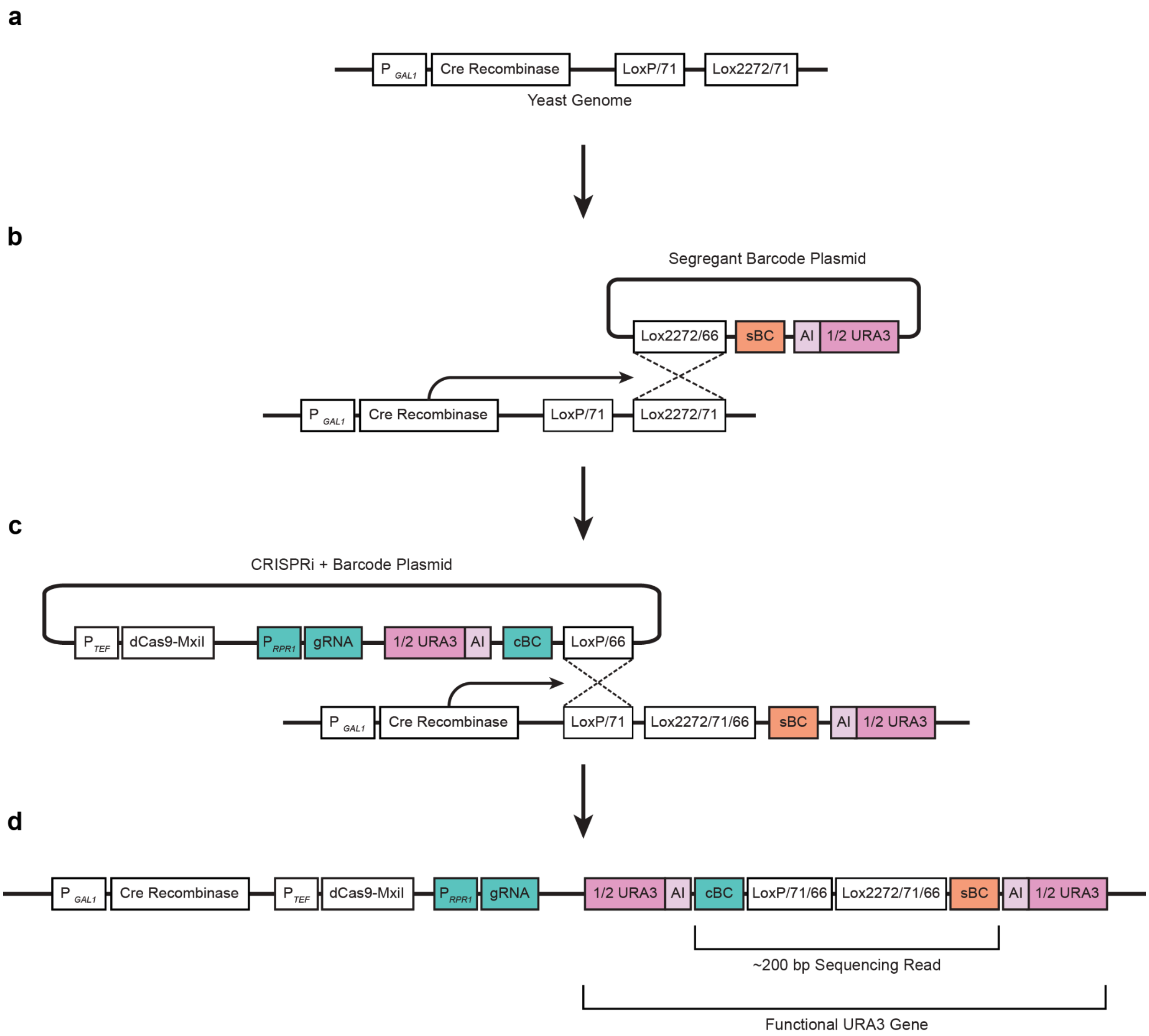

Supplemental Figure 3: Plasmid design and double-barcoding scheme. a) Genomic landing pad before integration of any plasmids. b) Integration of segregant barcode plasmid using the Lox2272 sites. A detailed visualization of this step is available in Supplemental Figure 1. c) Integration of barcoded CRISPRi plasmid library using the LoxP sites. d) Landing pad structure after CRISPRi plasmid integration. The *URA3* gene is only functional after CRISPRi plasmid integration, allowing for selection in SC - URA liquid media.

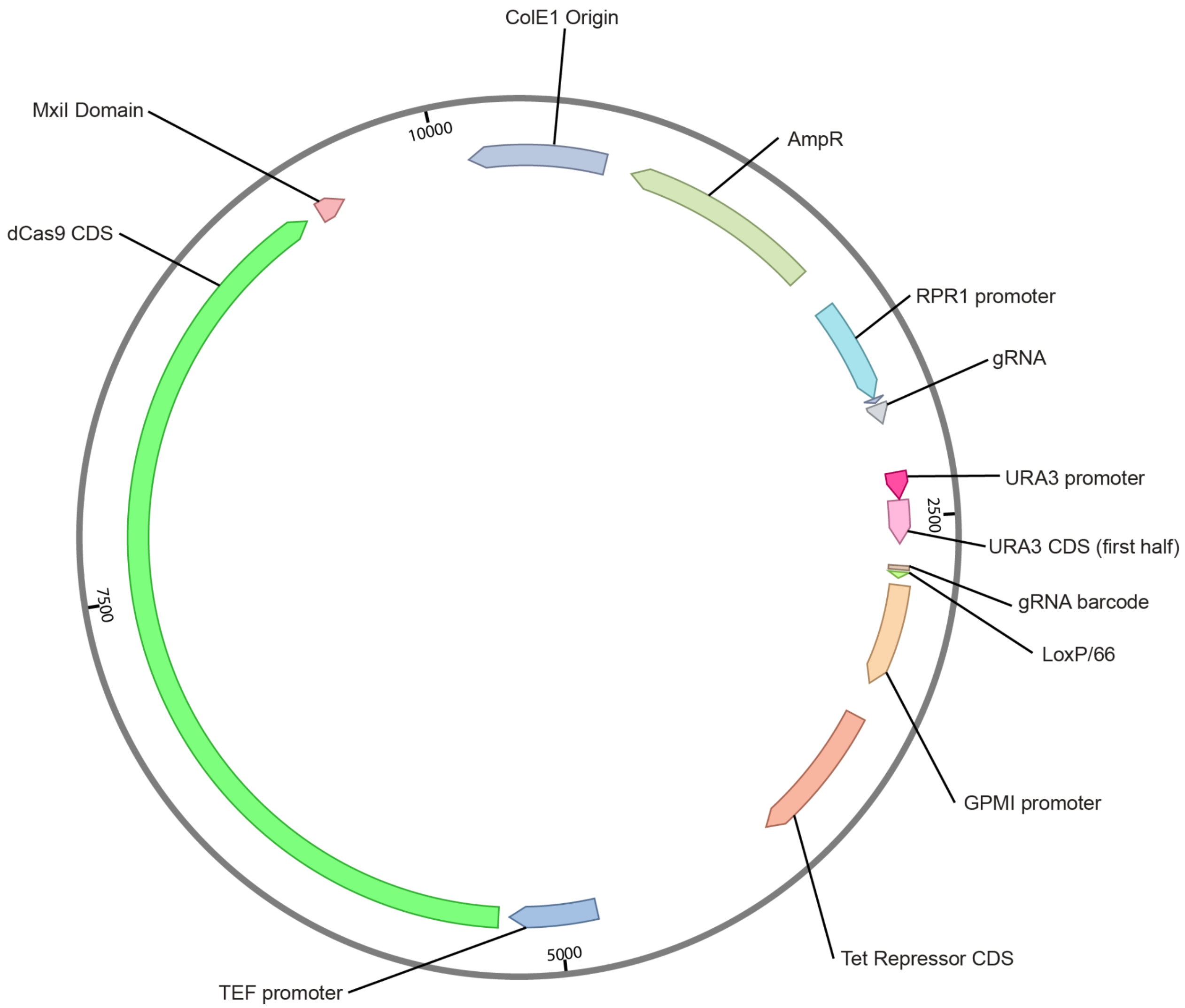

Supplemental Figure 4: CRISPRi plasmid map. This image was generated via an annotated plasmid map on Benchling (https://www.benchling.com/) and edited in Adobe Illustrator.

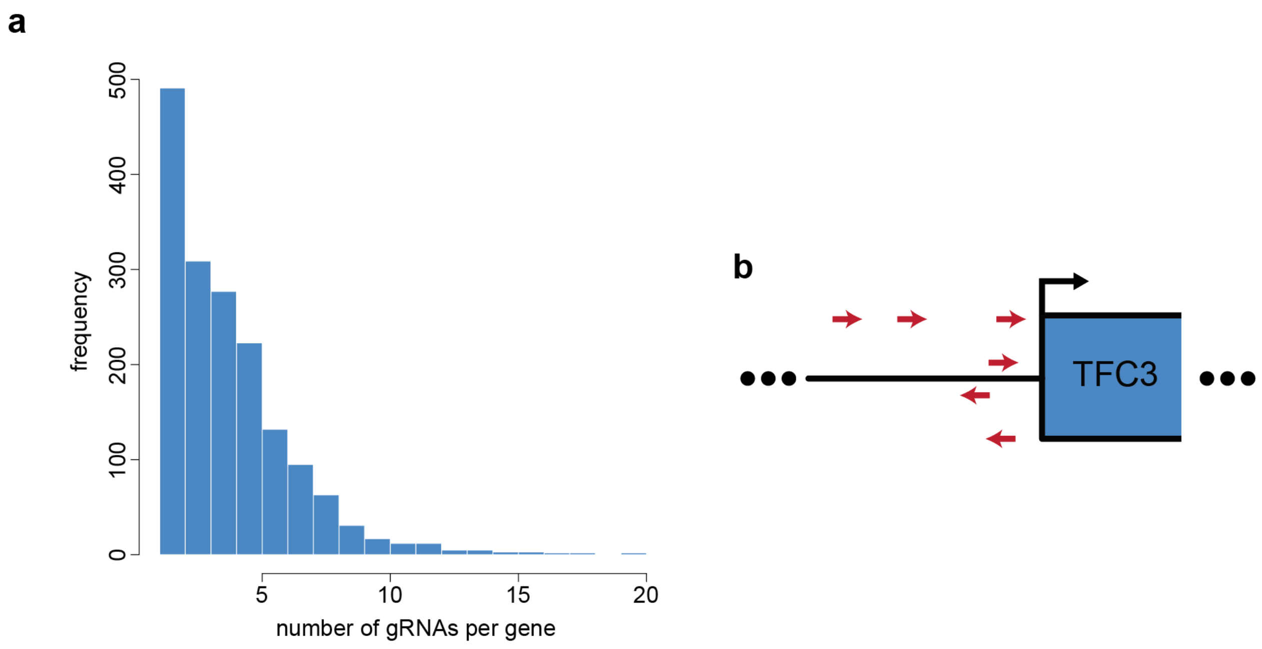

Supplemental Figure 5: Multiple replicate gRNAs target the same gene. a) Number of replicate gRNAs per gene (mean = 4.14, median = 4, sd = 2.65). b) An example of replicate gRNA positioning relative to a target gene. The blue box indicates the coding sequence of *TFC3/YAL001C*, with all gRNAs positioned around the promoter. Arrows indicate gRNA strand (plus/minus).

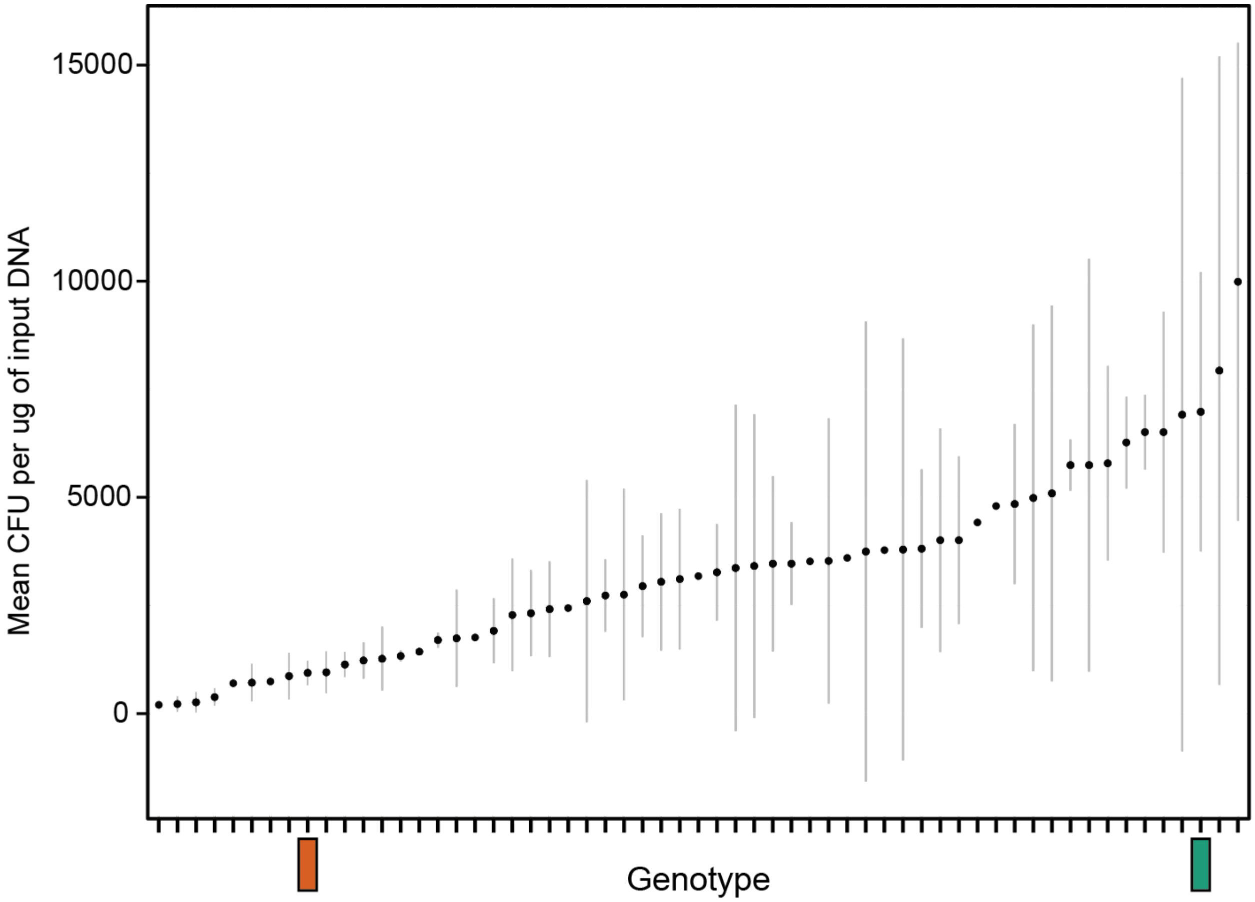

Supplemental Figure 6: Transformation efficiency across 58 random segregants. Segregants without error bars were not replicated. Dots represent means and error bars represent one standard deviation. The green bar indicates the BY parent, and the orange bar indicates the 3S parent.

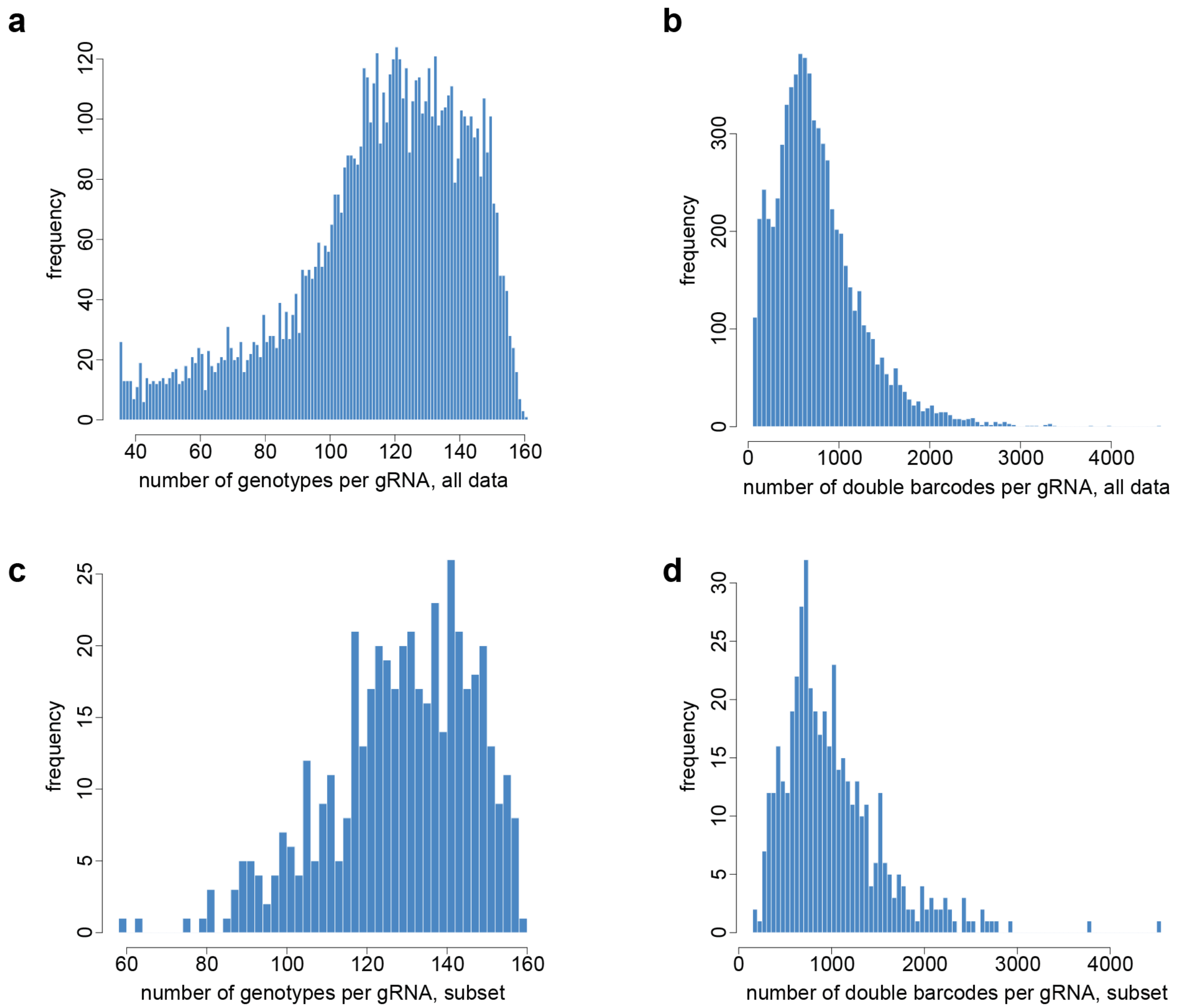

Supplemental Figure 7: Distribution of data per gRNA. a) Histogram of the number of genotypes per gRNA for all ~7,000 analyzed gRNAs (mean = 115.98, median = 120, sd = 26.84). b) Number of double barcodes per gRNA for all analyzed gRNAs (mean = 762.61, median = 674, sd = 474.65). c) Number of genotypes per gRNA for the 460 efficacious gRNAs used in downstream analyses (mean = 128.85, median = 131, sd = 18.14). d) Number of double barcodes per gRNA for gRNAs used in downstream analyses (mean = 1008.15, median = 892, sd = 549.26).

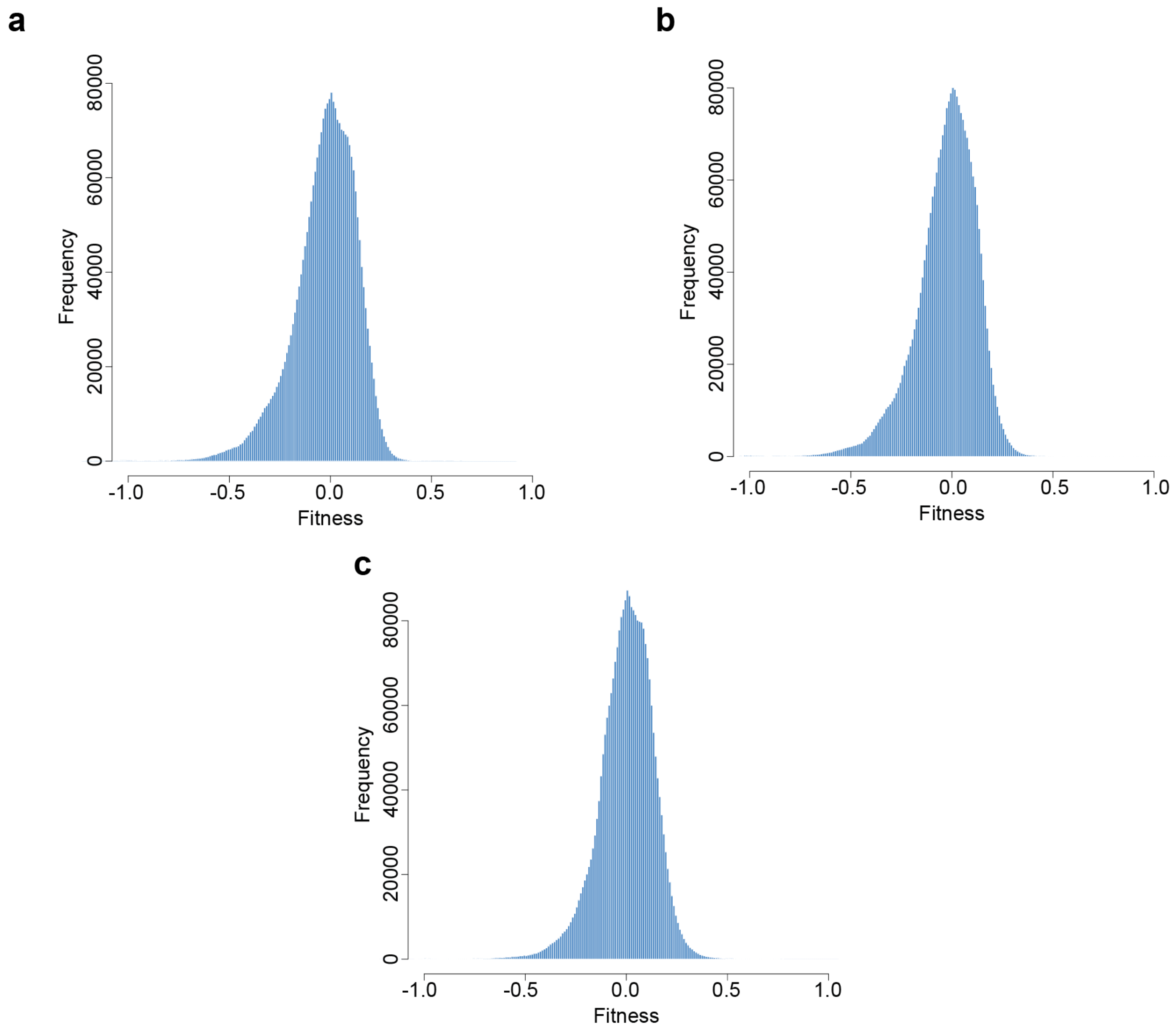

Supplemental Figure 8: Distribution of fitness values from each assay. a) Distribution of fitness values in ATC1. b) Distribution of fitness values in ATC2. c) Distribution of fitness values in CON.

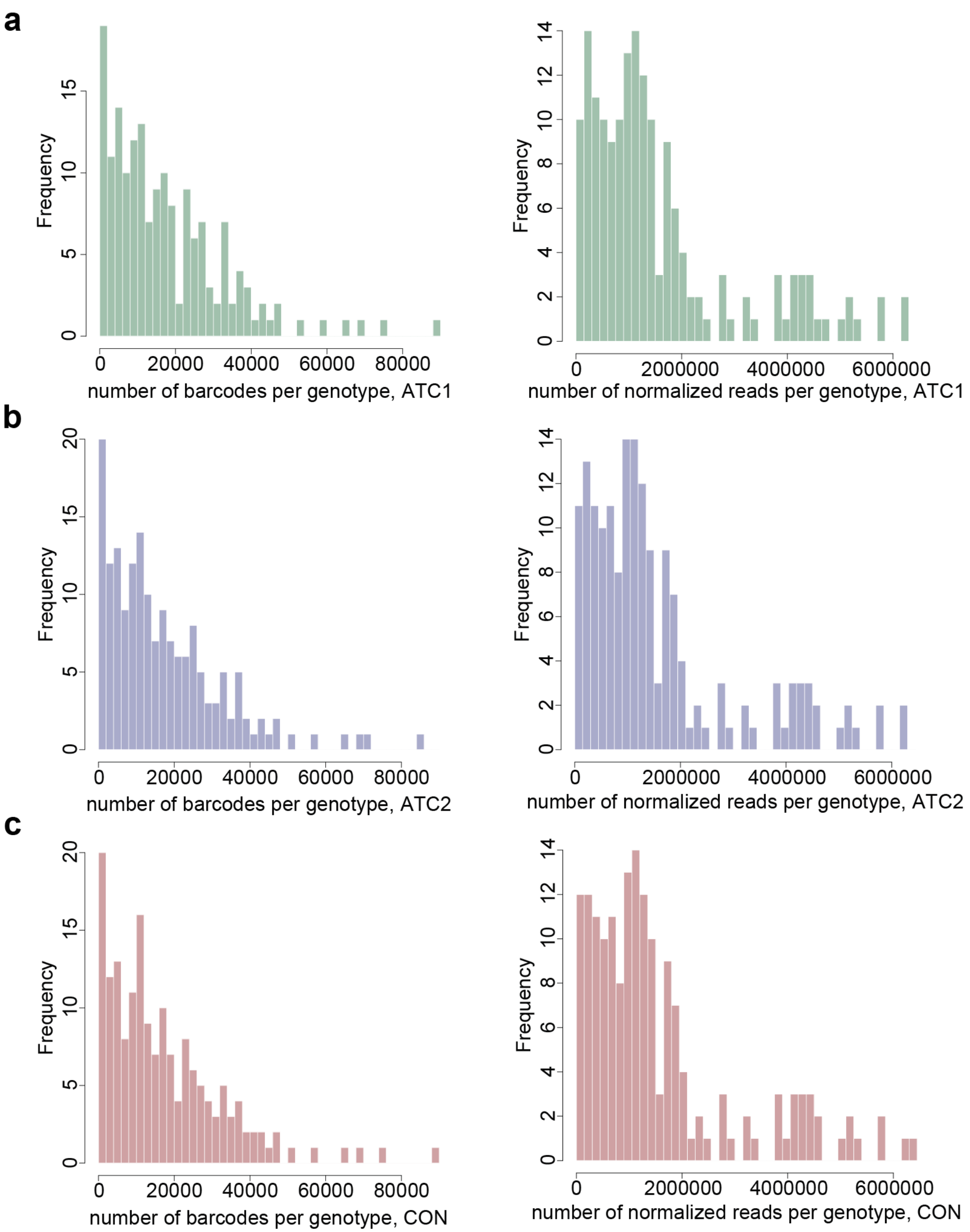

Supplemental Figure 9: Distribution of barcodes and normalized reads per genotype in each assay.

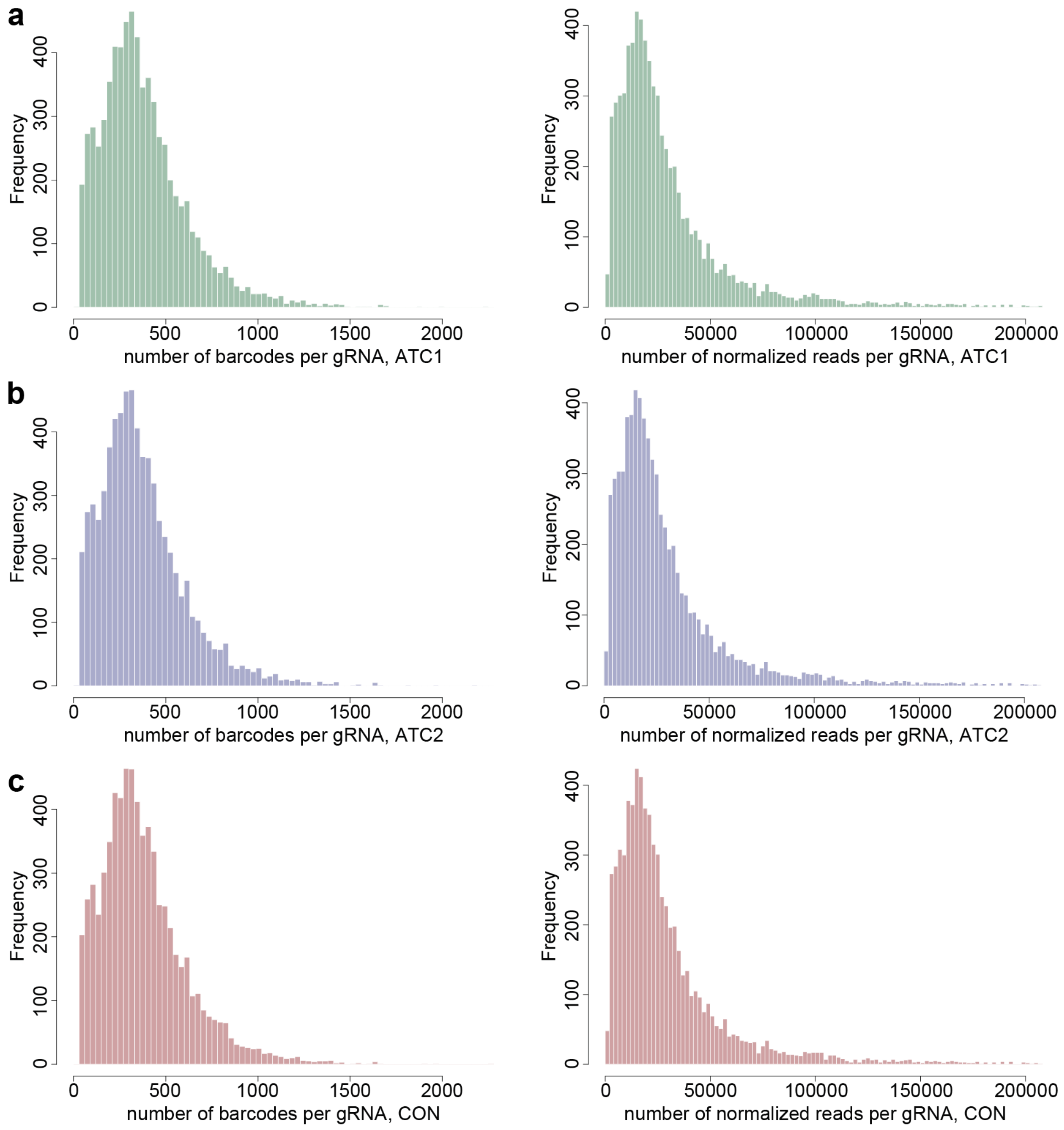

Supplemental Figure 10: Distribution of barcodes and normalized reads per gRNA in each assay. Normalized read histograms have extreme outliers removed for visibility (~1.5% of all gRNAs).

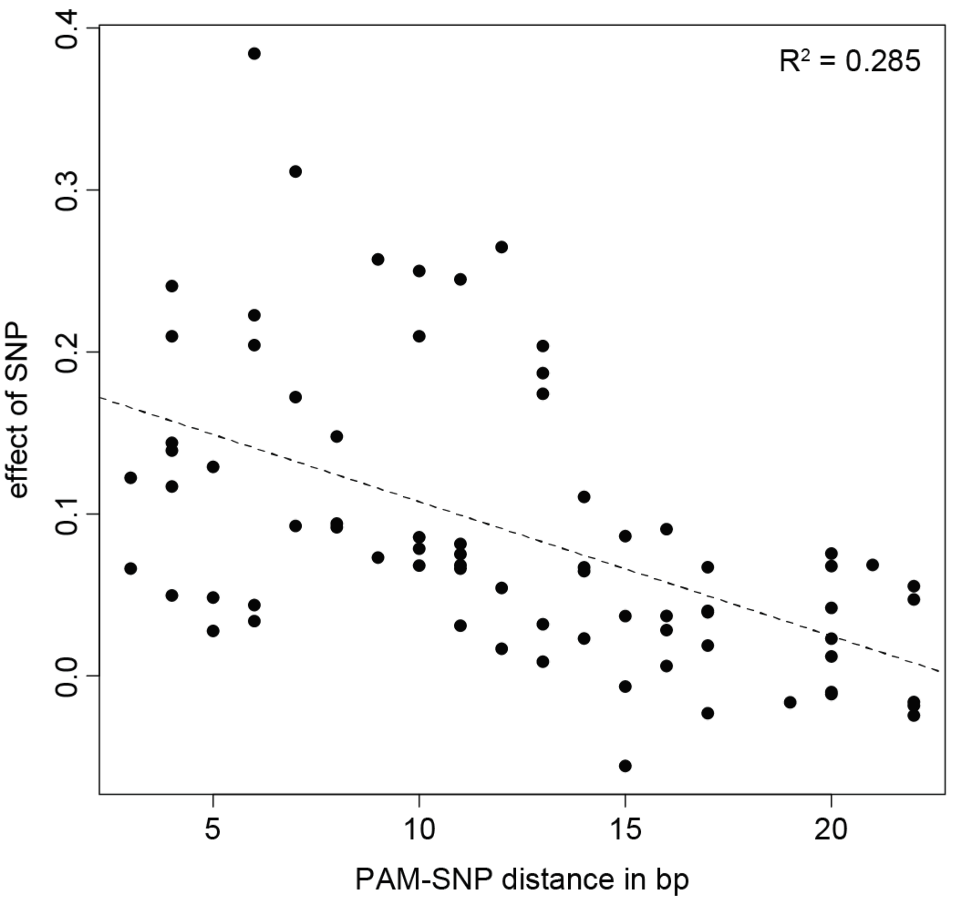

Supplemental Figure 11: XY plot between the effect of SNPs that directly overlap gRNA binding sites and the distance between the gRNA's PAM site and that SNP. The effect of the SNP is calculated as (effect of the gRNA in segregants with the BY allele) - (effect of the gRNA in segregants with the 3S allele). gRNAs perfectly match their targets in segregants with the BY allele. Simple linear regression R^2 = 0.285, pvalue = 9.9×10^{-7}.

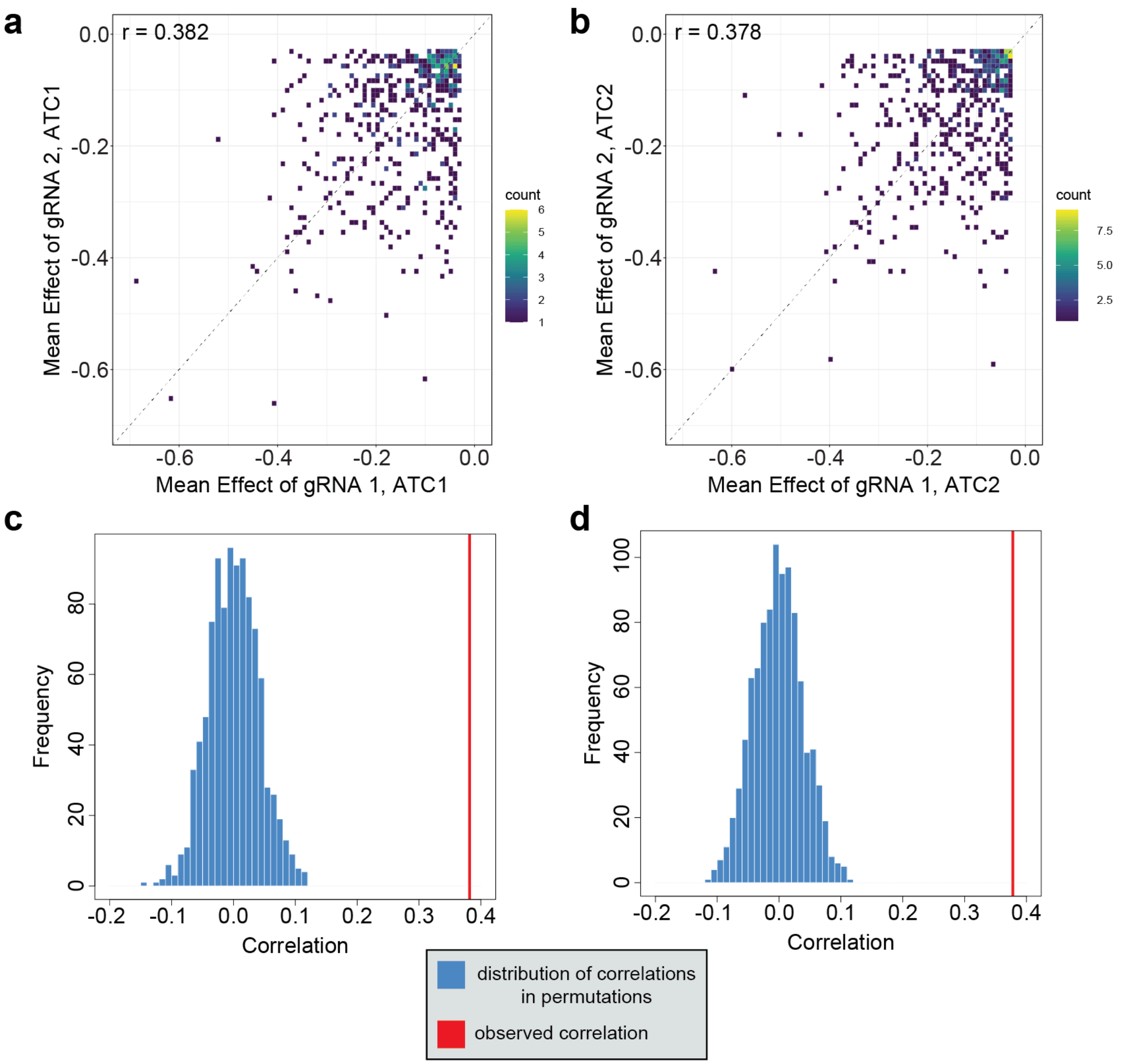

Supplemental Figure 12: Effects of gRNAs that target the same gene. gRNA effect was estimated by a mixed effects linear model (see Methods section 'Identification and Quantification of gRNA Effects'). If more than two gRNAs targeted the same gene, they were randomly assigned into pairs. The same subset of gRNAs was used for all following panels. a) X-Y plot showing the correlation between effects among gRNA pairs in the ATC1 condition (Pearson correlation = 0.382, pval = 5.61×10^{-19}). b) X-Y plot showing the correlation between effects among gRNA pairs in the ATC2 condition (Pearson correlation = 0.378, pval = 5.66×10^{-20}). c) Permutations for the correlation shown in (a). All gRNAs were randomly assigned into pairs, regardless of their targeted

gene, and the same Pearson correlation was calculated. This process was repeated 1000 times, with the resulting distribution of correlations shown in blue. The value from the original data set is shown by the red line. d) Permutations for the correlation shown in (b). All correlations were obtained via a two-sided test.

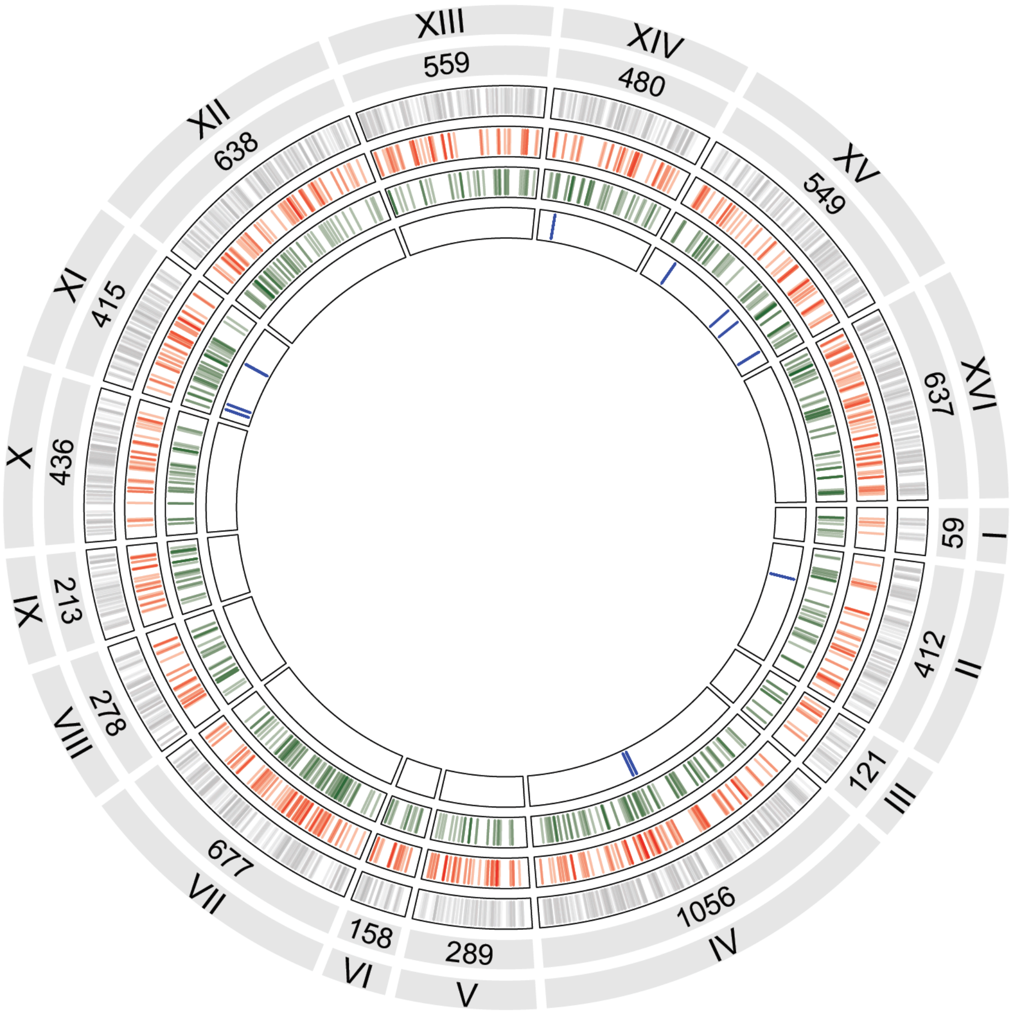

Supplemental Figure 13: Location of all gRNAs across the genome. Numbers show how many gRNAs in total are present on each chromosome. Gray lines indicate locations of gRNAs without mean effects. Red lines indicate locations of gRNAs with mean effects only. Green lines indicate gRNAs with background effects. Blue lines indicate control gRNAs.

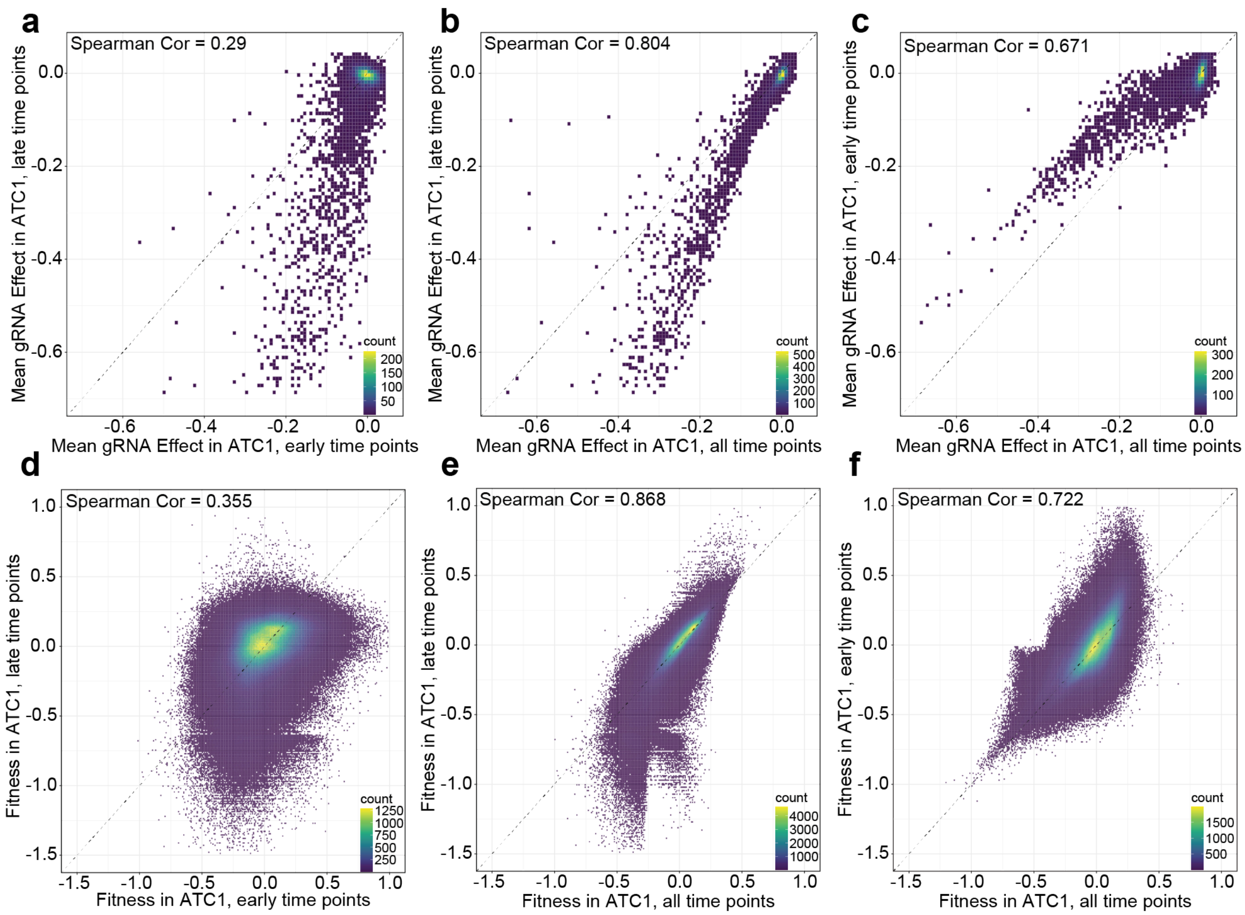

Supplemental Figure 14: Time dependence of mean gRNA effects and fitness estimates in ATC1. 'Early time points' refers to the first three time points in the fitness assay (T0, T1, and T2), 'late time points' refers to the final three (T2, T3, and T5), and 'all time points' refers to all five (T0, T1, T2, T3, and T5). Estimates are compared across time intervals for each lineage (for fitness estimates) or for each gRNA (for mean gRNA effects). a) Comparison of mean gRNA effects obtained from early time points and mean gRNA effects obtained from late time points. b) Mean gRNA effects across all and late time points. c) Mean gRNA effects across all and early time points. d) Fitness estimates across early and late time points. e) Fitness estimates across all and late time points. f) Fitness across between all and early time points.

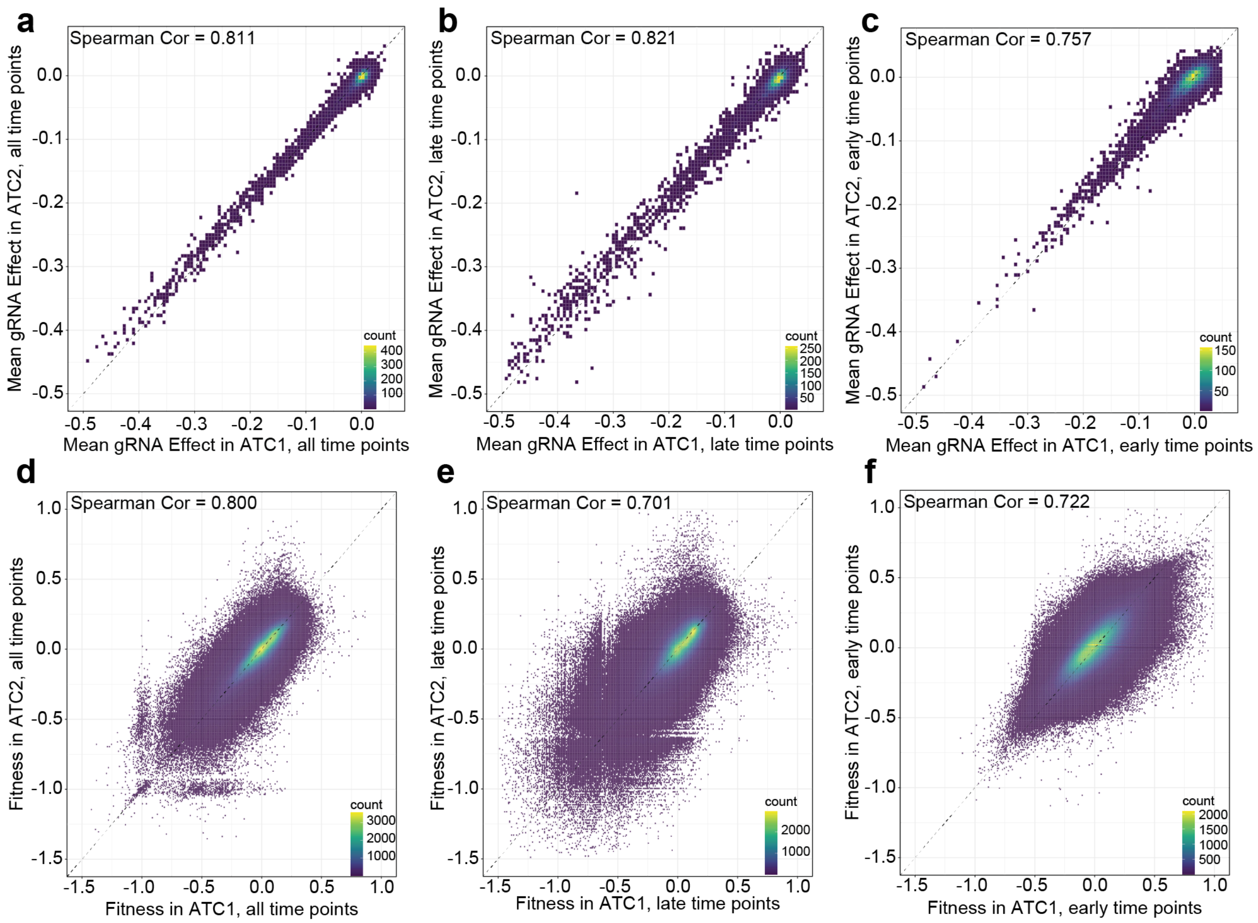

Supplemental Figure 15: Time dependence of reproducibility. 'Early time points' refers to the first three time points in the fitness assay (T0, T1, and T2), 'late time points' refers to the final three (T2, T3, and T5), and 'all time points' refers to all five (T0, T1, T2, T3, and T5). Estimates are compared across the two replicate ATC conditions for each lineage (for fitness estimates) or for each gRNA (for mean gRNA effects). a) Replicability of mean gRNA effects using all time points. b) Mean gRNA effects using late time points. c) Mean gRNA effects using early time points. d) Fitness estimates using all time points. e) Fitness estimates using late time points. f) Fitness estimates using early time points.

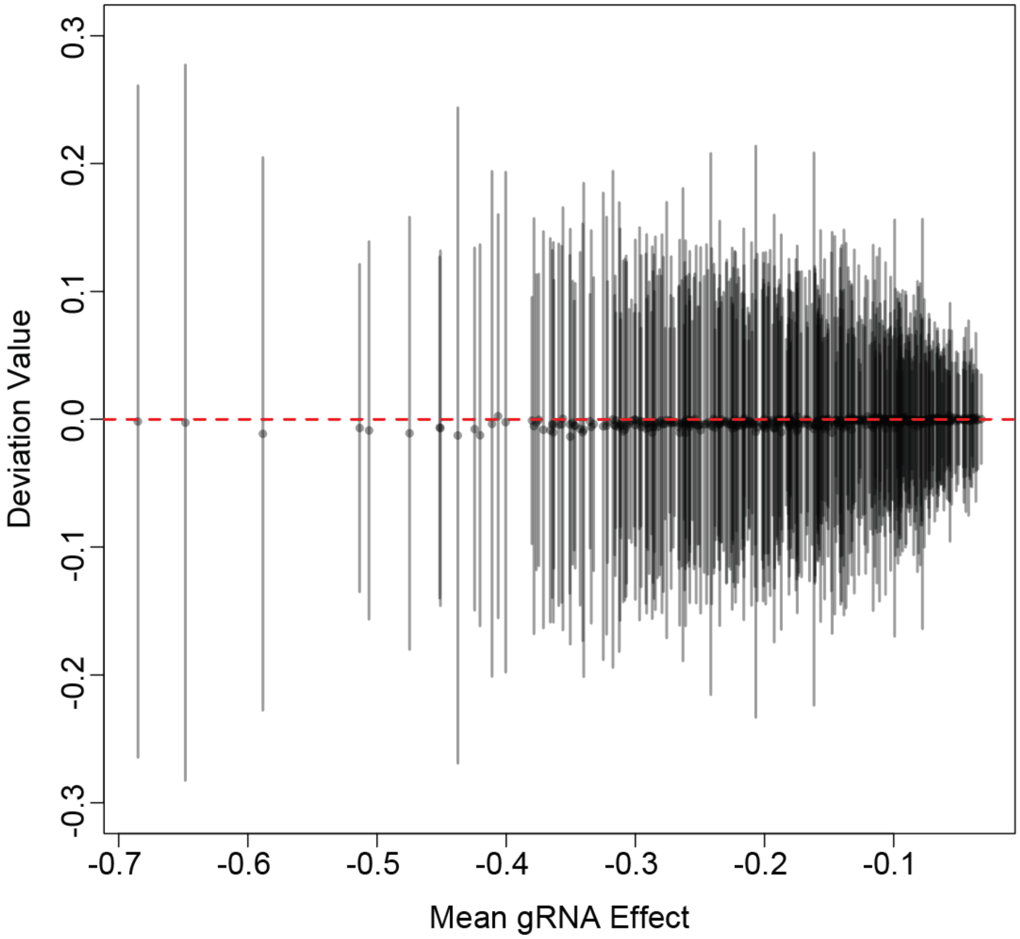

Supplemental Figure 16: X-Y plot between gRNA effect and all deviation values for that gRNA. Mean deviation value is shown by a point, with lines indicating two standard deviations. Each point represents a distinct gRNA-targeted gene with a background effect.

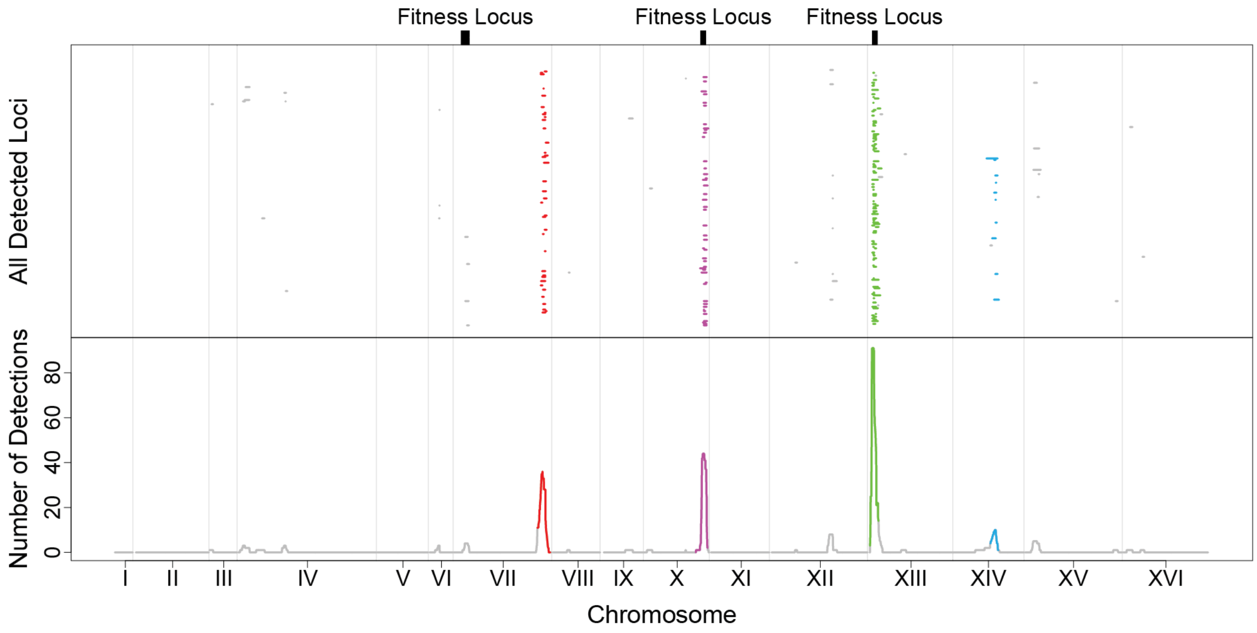

Supplemental Figure 17: Linkage mapping results on deviation values for the replicate experimental assay ATC2. Upper panel: Visualization of all 2x-$\log_{10}$(pval) drops for the gRNAs with significant peaks. Each row is a unique gRNA. Locations of major fitness loci are indicated by black bars. Lower panel: Number of overlapping 2x-$\log_{10}$(pval) drops at each nucleotide position along the genome. Regions where more intervals overlap than expected by chance (hubs) are shown in unique colors. Vertical lines indicate chromosome boundaries.

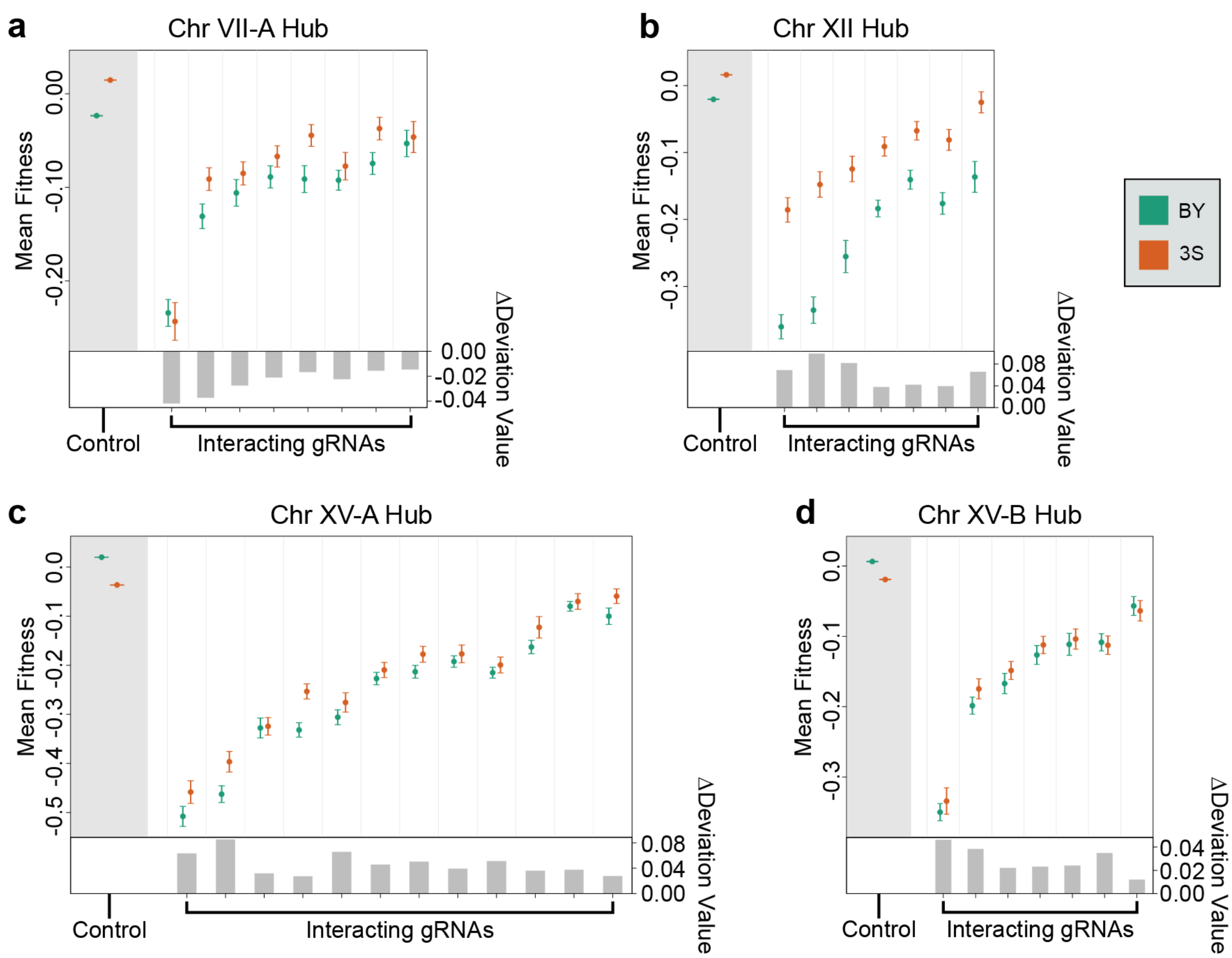

Supplemental Figure 18: Potential hubs below the significance threshold used during analysis. Hubs were categorized here if they passed the significance threshold of 9 overlapping confidence intervals in only one of the two conditions (ATC1 or ATC2), or if at least 5 overlapping confidence intervals were present in at least one condition. a) Effect of a locus near the 5' end of chromosome VII, at position 119852 and 39054 bp in width. This locus is distinct from the main Chr VII hub, and was detected as a significant locus during the linkage mapping on baseline fitness. This locus was detected only in ATC1. b) Average effect of the locus on chromosome XII, at position 662627 and 27942 bp in width. c) Average effect of the locus near the 5' end of chromosome XV, at position 147540 and 59773 bp in width. This locus was above the significance threshold only in ATC1. d) Average effect of the second locus on chromosome XV, at position 1011045 and 11091 in width. This locus was only detected in ATC1.

Supplemental Tables

Fitness Assay	Time Point	Total Raw Reads
ALL	0	460428002
ATC1	1	392520148
ATC1	2	399527009
ATC1	3	311268392
ATC1	5	365532111
ATC2	1	387400026
ATC2	2	354986473
ATC2	3	336666459
ATC2	5	376377113
CON	1	333135885
CON	2	349830935
CON	3	302795126
CON	5	401794794

Supplemental Table 1: Total raw reads used for fitness estimation. Reads were counted just before performing normalization and running PyFitSeq (see Methods section 'Fitness Estimation' for more detail).

gRNA Sequence	Chr	gRNA Start	gRNA End	PAM Position	SNP position	gRNA Effect
TGATGCATTCACTCTCAGTT	12	805700	805719	805697	805710	-0.0950996
GAACTCTAATACCTTTAGTT	2	691875	691894	691872	691880	-0.0624023
TTATCATAAACCAAGACCGG	7	999243	999262	999240	999249	-0.1315465
AAAAGAGTAACCTAGGAAAT	7	999219	999238	999216	999229	-0.0916477
AATATCACGATAGGACAAAT	12	694205	694224	694202	694211	-0.0356261
AGGCCTGAAAAATTTCAAAG	11	122400	122419	122422	122402	-0.0863247
GGAGAAAACTAAACGGGAAT	7	894494	894513	894516	894512	-0.1013647
AGGTTCCTGGTATCAAAGTG	7	767344	767363	767341	767344	-0.0415362
ATTCACATTCGAGGGGTTGC	4	238808	238827	238830	238826	-0.0738085
AAAACACGTTTGCATTGTTT	16	534884	534903	534906	534894	-0.1368684
TTAATCCGAGGTAATAGATT	2	124958	124977	124955	124959	-0.0926067
GATAATATCACCGTAGTTTC	13	99571	99590	99568	99579	-0.1239932
ATGCGTACAAGGAAGCGGGT	2	186645	186664	186642	186648	-0.0357824
TAATAATTCTCAATAGATAC	15	181592	181611	181614	181607	-0.1913804
GAACATCGATAAGTCGAATG	13	275896	275915	275918	275910	-0.09468
TATTTCTCTGTTAGATGCTC	15	633677	633696	633699	633695	-0.1050648
AGTTCCACTTTTCAGGCAGG	5	196820	196839	196842	196832	-0.0352892
GTTACCCTAATCTATTACCT	2	124950	124969	124972	124966	-0.2212353
TCGTAGGTTCGAATATCTCG	4	1398891	1398910	1398913	1398909	-0.0661702
TTCACATTCGAGGGGTTGCC	4	238809	238828	238831	238826	-0.0751744
CCAACTGCCTCAAAAAGGTA	9	261058	261077	261055	261061	-0.1216244
ACAGCAAAAAATAAGACTGC	15	802393	802412	802390	802400	-0.1444372
CCACTACATTTAGGTAGACG	13	901618	901637	901640	901630	-0.1003644

Supplemental Table 2: Table of gRNAs that are likely to be affected by SNPs near their binding location. The gRNAs shown all have significant background-dependent effects, a mapped QTL within 10 kb of the gRNA binding location, and a known BY/3S polymorphism at the gRNA binding location. More conservative thresholds were used when excluding gRNAs from analysis (see Methods section 'Linkage Mapping').

Time Point Set 1	Time Point Set 2	Pearson Correlation
T0-T1-T2-T3-T5	T0-T1-T2	0.691
T0-T1-T2-T3-T5	T0-T1-T2-T3	0.824
T0-T1-T2-T3-T5	T0-T1-T2-T3-T5-T7	0.933
T0-T1-T2-T3-T5	T1-T2-T3-T5	0.888
T0-T1-T2-T3-T5	T2-T3-T5-T7	0.704
T0-T1-T2-T3-T5	T1-T2-T3-T5-T7	0.837
T0-T1-T2	T0-T1-T2-T3	0.822
T0-T1-T2	T0-T1-T2-T3-T5-T7	0.613
T0-T1-T2	T1-T2-T3-T5	0.509
T0-T1-T2	T2-T3-T5-T7	0.244
T0-T1-T2	T1-T2-T3-T5-T7	0.469
T0-T1-T2-T3	T0-T1-T2-T3-T5-T7	0.767
T0-T1-T2-T3	T1-T2-T3-T5	0.678
T0-T1-T2-T3	T2-T3-T5-T7	0.495
T0-T1-T2-T3	T1-T2-T3-T5-T7	0.651
T0-T1-T2-T3-T5-T7	T1-T2-T3-T5	0.833
T0-T1-T2-T3-T5-T7	T2-T3-T5-T7	0.788
T0-T1-T2-T3-T5-T7	T1-T2-T3-T5-T7	0.906
T1-T2-T3-T5	T2-T3-T5-T7	0.729
T1-T2-T3-T5	T1-T2-T3-T5-T7	0.936
T2-T3-T5-T7	T1-T2-T3-T5-T7	0.819

Supplemental Table 3: Stability of fitness estimates across different subsets of time points. In order to test how the choice of time points affects PyFitSeq fitness estimates, the software was provided with a variable number of input time points from the ATC1 fitness assay. For each unique set of time points, a fitness estimate was generated for every lineage. The Pearson correlation was obtained by comparing the fitness estimates for each lineage across two sets of input time points via a two-sided test. The time point set ‘T0-T1-T2-T3-T5’ was the one used in this study. All p-values were below 2.2×10^{-16}.

gRNA Sequence	ORF	Chr	gRNA Position	Peak Start	Peak End	Peak Marker	-log10(pval)	condition
ATTAAAGTCATTTAGAGCAA	YBR237W	2	691848	678574	705106	681557	18.46	BOTH
AAAAGCACACCGTATAGAAA	YJR040W	10	507680	497086	545773	526954	14.05	BOTH
AAGGTTCCTGGTATCAAAGT	YGR138C	7	767345	748014	779852	766068	13.75	BOTH
TGAGTTCTAGCGGAAACCGC	YML098W	13	77154	27643	73993	32621	6.38	BOTH
TTTTGGCAAGCACGAATTGA	YJR111C	10	636901	642106	677358	660402	5.2	BOTH
GATACCAACCGATCCTTGCT	YGL167C	7	190876	154206	226152	175590	5.22	BOTH
TTCGCGTGGCATAACCCGGC	YML085C	13	99549	57144	104510	93860	6.95	BOTH
ATTTTGCTGGAAGGGCAATA	YLR347C	12	826667	785227	846812	807311	6.66	BOTH
AAGCTAATTAAGTGTAAACT	YKL028W	11	385663	367086	420060	394572	5.35	BOTH
ACTATCATAGGTATAAATAC	YKL137W	11	185849	164020	193550	184014	8.21	BOTH
ATGAGATTGAAACAATAACA	YJR112W	10	636945	641813	683971	655475	5.94	ATC2
TACTGGCCGCCGGCATGCGA	YJR002W	10	438744	415069	456781	434839	19.57	BOTH
AGAAGTATGGATGGTTCGAA	YGR186W	7	867710	846529	905027	869612	9.62	BOTH
ACCAAGACGCCACTACATTT	YMR314W	13	901609	899812	904006	900561	15.2	BOTH
TTTTATGAAGACTGGAGAAC	YJR040W	10	507664	496029	560037	545773	4.93	BOTH
GGCGGAAGGAGAAAGTTTCA	YDL139C	4	212162	176628	238826	222741	4.71	ATC1
TGTTAGTATTTAAGCTTATT	YMR287C	13	845438	824397	894039	855635	5.56	ATC1
TAAAAGCACACCGTATAGAA	YJR040W	10	507679	496029	544322	514236	10.77	ATC1
TATGAAGACTGGAGAACAGG	YJR040W	10	507661	496029	571066	531791	6.36	ATC1
GTTAAGCATTGTAAAATACC	YPL173W	16	222985	208747	244227	222598	4.5	ATC1
AAAGGTAAGAGATAATACTA	YOR159C	15	633624	571939	652034	585188	5.96	ATC1

Supplemental Table 4: Table of gRNAs with a mapped QTL near their binding location and without an overlapping polymorphism. The gRNAs shown all have background-dependent effects and a mapped QTL within 10 kb of the gRNA binding site. However, they lack any known polymorphism overlapping the gRNA binding site. All positions (gRNA position, interval start, interval end, and peak marker) are given in genome position. The width of the 2x-$\log_{10}$(pval) confidence interval is shown by the 'Interval Start' and 'Interval End' columns. The 'Condition' column describes which replicates a mapped QTL was detected in. Mapping was performed as described in the Methods section ‘Linkage mapping’.

Hub Chromosome	Number of gRNAs	Hub Marker Position	Peak Width (bp)
VII	38	979647	3371
X	25	646356	4918
XIII	76	43096	20469
XIV	18	470844	8643

Supplemental Table 5: Positional information about hubs. For each hub, the peak was defined as the marker of maximum significance across all gRNA interactions shown by a hub. The peak width was defined as the region where the highest number of confidence intervals across all gRNA interactions shown by a hub overlapped.

Supplemental Data

Supplemental Data 1: Barcode data for all segregants used in the fitness assay. Each row represents a unique segregant. The columns 'seg_name', 'geno_bc', and 'genotype' describe the segregant name, segregant barcode, and segregant genotype, respectively.

Supplemental Data 2: Genotype data for all segregants used in the fitness assay. Rows 'c', 'p', and 'gp' correspond to chromosome, position, and genome position. Each row is a polymorphic site among the segregants used in the assay. Each column contains the genotype data for a single segregant, with 0 indicating a BY allele and 1 indicating a 3S allele at that locus.

Supplemental Data 3: Data for all gRNAs used in the fitness assay. The 'guide', 'category', 'chrom', 'strand', 'start_gp', and 'end_gp' columns describe the gRNA sequence, gRNA library of origin, target chromosome, target strand, 5' genome position, and 3' genome position, respectively. The 'pam.mid.gp', 'ORF', and 'Gene_name' describe the midpoint of the PAM, the systematic name of the targeted ORF, and the standard name of the targeted ORF (if applicable). Control gRNAs show 'NA' under the ORF column.

Supplemental Data 4: Barcodes recovered per segregant. Each row is a unique segregant. The 'genotype' column provides the segregant name. The 'atc1_barcodes', 'atc2_barcodes', and 'con3_barcodes' describe the number of unique double barcodes recovered via sequencing for that segregant. The 'atc1_normalized_reads', 'atc2_normalized_reads', and 'con3_normalized_reads' describe the number of normalized reads recovered for that segregant in each fitness assay.

Supplemental Data 5: Barcodes recovered per gRNA. Each row is a unique gRNA, with the 'guide' column providing the gRNA name. The 'atc1_barcodes', 'atc2_barcodes', and 'con3_barcodes' describe the number of unique double barcodes

recovered via sequencing for that gRNA. The 'atc1_normalized_reads', 'atc2_normalized_reads', and 'con3_normalized_reads' describe the number of normalized reads recovered for that gRNA in each fitness assay. The 'number_of_genotypes' column shows how many segregants that gRNA appeared in.

Supplemental Data 6: Processed data from the ATC1 fitness assay. Each row represents a unique double barcode lineage. All double barcodes are linked to their respective segregant or gRNA. Each column contains the following information:
geno_bc: The genotype barcode sequence.
guide_bc: The gRNA barcode sequence.
T0: Normalized read count at time point 0 for this assay. Multiple adjacent columns follow this format (i.e. T1 for time point 1).
fitness: Fitness estimate from PyFitSeq software.
error: Reported error for the fitness estimate from PyFitSeq software.
Log_likelihood: Log likelihood score of the fitness estimate from PyFitSeq software.
Seg_name: Segregant name associated with the corresponding segregant barcode.
Genotype: Genotype name associated with that segregant.
Guide_seq: gRNA sequence associated with the gRNA barcode.
Gene_orf: The systematic name of the ORF targeted by the gRNA. This is 'NA' for control gRNAs.
Gene_name: The standard name of the ORF targeted by the gRNA, if applicable.
Guide_category: The plasmid library the gRNA originates from, with 'E' indicating the essential set used for analysis.

Supplemental Data 7: Processed data from the ATC2 fitness assay. Formatting is identical to file Supplemental Data 6.

Supplemental Data 8: Processed data from the CON fitness assay. Formatting is identical to file Supplemental Data 6.

Supplemental Data 9: Effects of gRNAs. Each row is a unique gRNA, with the 'guide', 'gene_orf', and 'gene_name' providing the gRNA sequence, systematic name of the targeted ORF, and standard name of the targeted ORF. The 'main.p' column is the p-value of the *gRNA* term from the linear model , and 'int.p' is the p-value of the *genotype:gRNA* interaction term. The 'main.fdr' and 'int.fdr' columns are the same p-values after Benjamini-Hochberg multiple testing correction. The 'nd.main.effect' columns shows if that gRNA had a mean effect three standard deviations below the median of the simulated distribution of neutral gRNAs. The 'guide.model.coef' column is the coefficient of the *gRNA* term extracted from the appropriate linear model. The 'number.of.points' and 'number.of.genos' columns show how many double barcodes and segregants were used to calculate gRNA effects, respectively.

Supplemental Data 10: Deviation values of gRNAs. Each column in this table is a unique gRNA, and each row is a unique segregant. The first column is composed of segregant names. Only gRNAs with background effects are present.

Supplemental Data 11: Results of linkage mapping on deviation values. Each row is a unique 2x-$\log_{10}$(pval) drop confidence interval. The 'chr', 'guide', and 'ORF' columns indicate the chromosome target, gRNA sequence, and systematic name of the targeted ORF. The 'peak.start' and 'peak.end' columns indicate the genome positions of the 5' end and 3' end of the confidence interval. The 'peak.marker', 'lod.score', 'adj_r2', and 'locus_coef' show the genome position of the peak marker, 2x-$\log_{10}$(pval), variance explained, and coefficient of the *locus* term, all from the linear model used at the peak marker locus. The columns 'total.geno.used' and 'locus_id' describe the number of unique segregants used when running the linear model, as well as the hub the interval is associated with (if any).

Supplemental Data 12: Genotype data for all segregants in the entire haploid panel. Formatting is identical to Supplemental Data 2.

Supplemental Data 13: Table of all SNPs present in the 3S strain, relative to S288C. Column 1 is the chromosome, column 2 is the SNP position, column 3 is the nucleotide in S288C, and column 4 is the nucleotide in 3S. The S288C reference differs from the BY strain by only roughly 125 SNPs.

Supplemental Data 14: Table of all linkages between gRNA barcodes and gRNA sequences. Column 1 is the gRNA barcode, column 2 is the gRNA sequence, column 3 is the targeted ORF, column 5 is the common name of the targeted gene, and column 5 is the classification of the targeted gene (essential or non-essential).
