# Supplementary material for: Genome-scale analysis of interactions between genetic perturbations and natural variation
Source: Nat Commun. 2024 May 18;15:4234. doi: 10.1038/s41467-024-48626-1 (PMC11102447; doi:10.1038/s41467-024-48626-1)
Supplement: Supplementary file 3 — Description of Additional Supplementary Files [file 41467_2024_48626_MOESM3_ESM.pdf]

Description of Additional Supplementary Files

Supplemental Data 1: Barcode data for all segregants used in the fitness assay. Each row represents a unique segregant. The columns 'seg_name', 'geno_bc', and 'genotype' describe the segregant name, segregant barcode, and segregant genotype, respectively.

Supplemental Data 2: Genotype data for all segregants used in the fitness assay. Rows 'c', 'p', and 'gp' correspond to chromosome, position, and genome position. Each row is a polymorphic site among the segregants used in the assay. Each column contains the genotype data for a single segregant, with 0 indicating a BY allele and 1 indicating a 3S allele at that locus.

Supplemental Data 3: Data for all gRNAs used in the fitness assay. The 'guide', 'category', 'chrom', 'strand', 'start_gp', and 'end_gp' columns describe the gRNA sequence, gRNA library of origin, target chromosome, target strand, 5’ genome position, and 3’ genome position, respectively. The 'pam.mid.gp', 'ORF', and 'Gene_name' describe the midpoint of the PAM, the systematic name of the targeted ORF, and the standard name of the targeted ORF (if applicable). Control gRNAs show 'NA' under the ORF column.

Supplemental Data 4: Barcodes recovered per segregant. Each row is a unique segregant. The 'genotype' column provides the segregant name. The 'atc1_barcodes', 'atc2_barcodes', and 'con3_barcodes' describe the number of unique double barcodes recovered via sequencing for that segregant. The 'atc1_normalized_reads', 'atc2_normalized_reads', and 'con3_normalized_reads' describe the number of normalized reads recovered for that segregant in each fitness assay.

Supplemental Data 5: Barcodes recovered per gRNA. Each row is a unique gRNA, with the 'guide' column providing the gRNA name. The 'atc1_barcodes', 'atc2_barcodes', and 'con3_barcodes' describe the number of unique double barcodes

recovered via sequencing for that gRNA. The 'atc1_normalized_reads', 'atc2_normalized_reads', and 'con3_normalized_reads' describe the number of normalized reads recovered for that gRNA in each fitness assay. The 'number_of_genotypes' column shows how many segregants that gRNA appeared in.

Supplemental Data 6: Processed data from the ATC1 fitness assay. Each row represents a unique double barcode lineage. All double barcodes are linked to their respective segregant or gRNA. Each column contains the following information:
geno_bc: The genotype barcode sequence.
guide_bc: The gRNA barcode sequence.
T0: Normalized read count at time point 0 for this assay. Multiple adjacent columns follow this format (i.e. T1 for time point 1).
fitness: Fitness estimate from PyFitSeq software.
error: Reported error for the fitness estimate from PyFitSeq software.
Log_likelihood: Log likelihood score of the fitness estimate from PyFitSeq software.
Seg_name: Segregant name associated with the corresponding segregant barcode.
Genotype: Genotype name associated with that segregant.
Guide_seq: gRNA sequence associated with the gRNA barcode.
Gene_orf: The systematic name of the ORF targeted by the gRNA. This is 'NA' for control gRNAs.
Gene_name: The standard name of the ORF targeted by the gRNA, if applicable.
Guide_category: The plasmid library the gRNA originates from, with 'E' indicating the essential set used for analysis.

Supplemental Data 7: Processed data from the ATC2 fitness assay. Formatting is identical to file Supplemental Data 6.

Supplemental Data 8: Processed data from the CON fitness assay. Formatting is identical to file Supplemental Data 6.

Supplemental Data 9: Effects of gRNAs. Each row is a unique gRNA, with the 'guide', 'gene_orf', and 'gene_name' providing the gRNA sequence, systematic name of the targeted ORF, and standard name of the targeted ORF. The 'main.p' column is the p-value of the *gRNA* term from the linear model , and 'int.p' is the p-value of the *genotype:gRNA* interaction term. The 'main.fdr' and 'int.fdr' columns are the same p-values after Benjamini-Hochberg multiple testing correction. The 'nd.main.effect' columns shows if that gRNA had a mean effect three standard deviations below the median of the simulated distribution of neutral gRNAs. The 'guide.model.coef' column is the coefficient of the *gRNA* term extracted from the appropriate linear model. The 'number.of.points' and 'number.of.genos' columns show how many double barcodes and segregants were used to calculate gRNA effects, respectively.

Supplemental Data 10: Deviation values of gRNAs. Each column in this table is a unique gRNA, and each row is a unique segregant. The first column is composed of segregant names. Only gRNAs with background effects are present.

Supplemental Data 11: Results of linkage mapping on deviation values. Each row is a unique 2x-$\log_{10}$(pval) drop confidence interval. The 'chr', 'guide', and 'ORF' columns indicate the chromosome target, gRNA sequence, and systematic name of the targeted ORF. The 'peak.start' and 'peak.end' columns indicate the genome positions of the 5’ end and 3’ end of the confidence interval. The 'peak.marker', 'lod.score', 'adj_r2', and 'locus_coef' show the genome position of the peak marker, 2x-$\log_{10}$(pval), variance explained, and coefficient of the *locus* term, all from the linear model used at the peak marker locus. The columns 'total.geno.used' and 'locus_id' describe the number of unique segregants used when running the linear model, as well as the hub the interval is associated with (if any).

Supplemental Data 12: Genotype data for all segregants in the entire haploid panel. Formatting is identical to Supplemental Data 2.

Supplemental Data 13: Table of all SNPs present in the 3S strain, relative to S288C. Column 1 is the chromosome, column 2 is the SNP position, column 3 is the nucleotide in S288C, and column 4 is the nucleotide in 3S. The S288C reference differs from the BY strain by only roughly 125 SNPs.

Supplemental Data 14: Table of all linkages between gRNA barcodes and gRNA sequences. Column 1 is the gRNA barcode, column 2 is the gRNA sequence, column 3 is the targeted ORF, column 5 is the common name of the targeted gene, and column 5 is the classification of the targeted gene (essential or non-essential).
