# Supplementary material for: Genome-scale analysis of interactions between genetic perturbations and natural variation
Source: Nat Commun. 2024 May 18;15:4234. doi: 10.1038/s41467-024-48626-1 (PMC11102447; doi:10.1038/s41467-024-48626-1)
Supplement: Supplementary file 18 — Reporting Summary [file 41467_2024_48626_MOESM18_ESM.pdf]

nature portfolio

Corresponding author(s): Ehrenreich, IM
Levy, SF

Last updated by author(s): April 15, 2024

Reporting Summary

Nature Portfolio wishes to improve the reproducibility of the work that we publish. This form provides structure for consistency and transparency in reporting. For further information on Nature Portfolio policies, see our Editorial Policies and the Editorial Policy Checklist.

Statistics

For all statistical analyses, confirm that the following items are present in the figure legend, table legend, main text, or Methods section.

n/a	Confirmed	
☐	☒	The exact sample size (*n*) for each experimental group/condition, given as a discrete number and unit of measurement
☐	☒	A statement on whether measurements were taken from distinct samples or whether the same sample was measured repeatedly
☐	☒	The statistical test(s) used AND whether they are one- or two-sided<br>*Only common tests should be described solely by name; describe more complex techniques in the Methods section.*
☐	☒	A description of all covariates tested
☐	☒	A description of any assumptions or corrections, such as tests of normality and adjustment for multiple comparisons
☐	☒	A full description of the statistical parameters including central tendency (e.g. means) or other basic estimates (e.g. regression coefficient) AND variation (e.g. standard deviation) or associated estimates of uncertainty (e.g. confidence intervals)
☐	☒	For null hypothesis testing, the test statistic (e.g. *F*, *t*, *r*) with confidence intervals, effect sizes, degrees of freedom and *P* value noted<br>*Give P values as exact values whenever suitable.*
☒	☐	For Bayesian analysis, information on the choice of priors and Markov chain Monte Carlo settings
☐	☒	For hierarchical and complex designs, identification of the appropriate level for tests and full reporting of outcomes
☐	☒	Estimates of effect sizes (e.g. Cohen's *d*, Pearson's *r*), indicating how they were calculated

Our web collection on statistics for biologists contains articles on many of the points above.

Software and code

Policy information about availability of computer code

Data collection	No software was used for data collection.
Data analysis	Python and R were used to perform analyses in this study. In Python, we used Bartender, PyFitSeq, and rapidfuzz (v2.13.0) software and packages. In R, we used nlme (v3.1.160) and sommer (v4.3.2) packages for analysis, as well as ggplot2 (v3.4.3) and circlize (v0.4.15) packages for data visualization. We also supplied our code in the supplement, which is available via figshare https://figshare.com/s/c4c90d476ae701a6cf16.

For manuscripts utilizing custom algorithms or software that are central to the research but not yet described in published literature, software must be made available to editors and reviewers. We strongly encourage code deposition in a community repository (e.g. GitHub). See the Nature Portfolio guidelines for submitting code & software for further information.

Data

Policy information about availability of data

All manuscripts must include a data availability statement. This statement should provide the following information, where applicable:

- Accession codes, unique identifiers, or web links for publicly available datasets
- A description of any restrictions on data availability
- For clinical datasets or third party data, please ensure that the statement adheres to our policy

The barcode sequencing data generated in this study have been deposited in the NCBI Sequence Read Archive Bioproject under accession code PRJNA986287

[https://www.ncbi.nlm.nih.gov/bioproject/PRJNA986287]. Data used to generate figures are available through the Source Data file. Supplemental data files are available through Figshare at: https://figshare.com/s/c4c90d476ae701a6cf16. The S288c reference genome data used in this study is available at http://sgd-archive.yeastgenome.org/sequence/S288C_reference/. The data used for SAFE analysis via TheCellMap is available at https://thecellmap.org/.

Research involving human participants, their data, or biological material

Policy information about studies with human participants or human data. See also policy information about sex, gender (identity/presentation), and sexual orientation and race, ethnicity and racism.

Reporting on sex and gender	N/A
Reporting on race, ethnicity, or other socially relevant groupings	N/A
Population characteristics	N/A
Recruitment	N/A
Ethics oversight	N/A

Note that full information on the approval of the study protocol must also be provided in the manuscript.

Field-specific reporting

Please select the one below that is the best fit for your research. If you are not sure, read the appropriate sections before making your selection.

☒ Life sciences ☐ Behavioural & social sciences ☐ Ecological, evolutionary & environmental sciences

For a reference copy of the document with all sections, see nature.com/documents/nr-reporting-summary-flat.pdf

Life sciences study design

All studies must disclose on these points even when the disclosure is negative.

Sample size	Our sample size was based on all of the transformed strains we had available. All available strains were included in our study.
Data exclusions	We used nearly all generated data in our analyses. Only sequencing reads that could not be associated with known barcodes in our study were excluded.
Replication	We performed our experimental assay twice independently and also performed a control assay. All attempts at replication were successful.
Randomization	All strains in our study were grown in a common pool with all strains biologically replicated in the pool. Thus, strains were not allocated into separate experimental groups, which is an advantage of our method.
Blinding	As all samples were grown in a single common pool, no blinding was necessary in this study.

Reporting for specific materials, systems and methods

We require information from authors about some types of materials, experimental systems and methods used in many studies. Here, indicate whether each material, system or method listed is relevant to your study. If you are not sure if a list item applies to your research, read the appropriate section before selecting a response.

Materials & experimental systems

n/a	Involved in the study	
☒	☐	Antibodies
☒	☐	Eukaryotic cell lines
☒	☐	Palaeontology and archaeology
☒	☐	Animals and other organisms
☒	☐	Clinical data
☒	☐	Dual use research of concern
☒	☐	Plants

Methods

n/a	Involved in the study	
☒	☐	ChIP-seq
☒	☐	Flow cytometry
☒	☐	MRI-based neuroimaging

Plants

Seed stocks	N/A
Novel plant genotypes	N/A
Authentication	N/A
